# The Influence of Selected Fillers on the Functional Properties of Polycarbonate Dedicated to 3D Printing Applications

**DOI:** 10.3390/polym16050592

**Published:** 2024-02-21

**Authors:** Katarzyna Bulanda, Mariusz Oleksy, Rafał Oliwa

**Affiliations:** Department of Polymer Composites, Faculty of Chemistry, Rzeszow University of Technology, Al. Powstańców Warszawy 6, 35-959 Rzeszów, Poland; oliwa@prz.edu.pl

**Keywords:** additive manufacturing, 3D printing, MEM, polymer composites, PC, fillers, bentonite, silica

## Abstract

Additive manufacturing is still the fastest-developing technology in the modern world. Three-dimensional printing has become popular due to the method’s numerous advantages, such as its short time and low cost, compared to conventional methods such as injection molding. Therefore, the demand for new materials and material systems that will be characterized by the desired functional properties is clearly growing. As part of this work, work was carried out on the development and preparation of new polymer composites dedicated to 3D printing applications, especially in FDM/FFF/MEM technologies. The influence of the content and amount of fillers, such as silica modified with alumina (S) and bentonite modified with a quaternary ammonium salt (B), on the functional properties of a commercially available fiber made of traditional plastic, such as polycarbonate, obtained in the form of a filament (PCF), was determined. It was found that the addition of B significantly increased the fluidity of the polymer, the introduction of a filler in the amount of 1.5% allowed to obtain a result that was 6% higher compared to PCF (16.8 g/10 min), while the amount of 3% was 20% higher. The obtained mass melt flow rate (MFR) results were confirmed by determining the viscosity of the produced polymer composites. Satisfactory results of mechanical properties were obtained, including the following: it was found that the introduced modified fillers increased the elasticity of the material. The introduction of modified silica resulted in a reduction in Young’s modulus by 10.02% at the content of 0.5% S and at 1% S by 8.64% compared to the polymer. The introduced modified filler S significantly increased the thermostability of polycarbonate (T_5%_ equal to 449 °C) by 23 °C for PCF/0.5% S and 14 °C for PCF/1% S, respectively. The SEM and WAXS results confirmed the appropriate dispersion of the fillers in the polymer matrix, which indicates well-selected conditions for the homogenization process of the components and the subsequent production of samples. Detailed characterization of the influence of selected fillers on the functional properties of the polymer matrix-polycarbonate allowed for an increase in the range of polymer composites and their use in rapid prototyping technologies, as well as supplementing the literature on databases regarding the characteristics of the obtained materials.

## 1. Introduction

Currently, there is a continuous development of rapid prototyping techniques and, consequently, the use of a number of materials in technology. In the material extrusion method, special emphasis is placed on the development of innovative polymer materials, which are introduced into 3D printers in the form of a filament. Examples of this printing method are techniques known commercially as fused deposition modeling (FDM), fused fiber fabrication (FFF) and melt extrusion manufacturing (MEM).

FDM/FFF/MEM technologies enable the production of parts by applying polymer material in layers. The material in the form of a filament is heated to the melting point of the material, then extruded through a nozzle and placed on the printer’s work table in such a way that subsequent layers bond with the previously applied ones [1,2,3]. When obtaining a single layer, the head (engine, melting coil and nozzle) moves in the XY plane over the detail. By lowering the working platform or raising the head, it is possible to apply another layer [4]. The quality of processed elements obtained using FDM/FFF/MEM depends mainly on the careful selection of the so-called process variables. Therefore, it is very important to know the technology parameters that have a very large impact on the quality of the obtained parts [1,2,5,6,7].

Materials intended for use in 3D printing are characterized by various properties, certain advantages and disadvantages as well as different requirements regarding their processing parameters, such as a specific range of application temperatures. Most often, the material is selected taking into account primarily the type of available equipment because printers operating in FDM/FFF/MEM technologies do not process certain types of raw materials; they are selected in terms of their functional properties (mechanical, physicochemical) and their adaptation to the final purpose model [8,9,10,11].

Auxiliary agents introduced into plastics in the process of polymer synthesis or at the stage of their processing perform various functions, such as modifying the rheological, mechanical and functional properties of the material. The modification of plastics is generally a more economical process in relation to the need to develop and synthesize new monomers and polymers for selected applications [12].

The physical modification of thermoplastics is a simpler and cheaper method in comparison with chemical modification. It involves changing the physicochemical properties of the polymer by introducing additional ingredients that lead to changes in the composition or by the action of physical factors, such as ultrasound or thermal energy. However, the chemical modification of thermoplastics involves changing the chemical composition of macromolecules and, thus, changing the properties of the material. Modification may occur during, for example, intramolecular cyclization, as well as during grafting, reduction and oxidation reactions [12].

In order to change the properties of the material, especially for 3D printing applications, physical modification is used. For this purpose, various types of additives are introduced into the polymer matrix. A properly conducted homogenization process allows for good dispersion of auxiliary agents in the material and the final stage allows the material to be extruded in the form of a fiber of a specific diameter. Typical auxiliaries used for thermoplastics are fillers, stabilizers, plasticizers, dyes, release agents, pigments, flame retardants and antistatic agents. Fragrances and biostabilizers can also be added to the polymer matrix.

By modifying polymers dedicated to 3D printing applications, polymer composites can be obtained or, when the component has at least one dimension on the nanometer scale, nanocomposites. Polymer composites are created by introducing organic, inorganic particles or hybrid systems into the polymer matrix, including inorganic–organic. The introduced particles can be in the form of powder (e.g., metal particles), plates (e.g., aluminosilicates, especially the well-known montmorillonite) and fibers or rods (e.g., carbon fibers or pipes) [13].

In order to increase the popularity of 3D printing, we should strive to achieve the most affordable prices for filaments. The cost of 3D printing materials, including thermoplastics, can be reduced by adding fillers, which are essentially cheap materials. The introduced additives can have a positive impact on the increase in dimensional stability after solidification but also on the increase in bending stiffness. However, they also cause an increase in density and a decrease in the tensile strength of the obtained composites. To some extent, hard filler particles can affect the service life of 3D printers, increasing their depreciation cost [9].

To modify thermoplastics, many inexpensive fillers are standardly used, such as inorganic silica, which, among commonly available auxiliaries, is often used due to its many advantages, including a lack of reactivity and high chemical and thermal stability. Silica or silicon dioxide may be anhydrous or hydrated. Among the mineral fillers, the most active is anhydrous silica [14]. The literature confirms that the addition of silica as a strengthening agent modifies the properties of material-tensile strength and elastic modulus [15,16,17]. The literature reveals several works in which scientists introduced silica into thermoplastic materials, thus obtaining filaments for 3D printing. Silica is used as a filler in materials dedicated to additive manufacturing in order to control, above all, the viscosity of the material in order to ensure the proper course of the extrusion process [18]. The filler was introduced, among others, into PLA [19] in amounts ranging from 0% to 8% by weight, obtaining satisfactory mechanical and tribological properties. The influence of the filler on the hygroscopicity of the PLA fiber was also examined [20]. Silanol-treated nanosilica successfully improved the PLA matrix, especially when introducing 1% silica by weight, where the greatest improvement was achieved in terms of reducing hygroscopicity and, at the same time, increasing tensile properties relative to PLA. The article [15] examined the influence of a mixture of silica and waste PLA on the mechanical properties. The result indicated that increasing the silica composition (at a concentration of 10 wt.%) resulted in an increase in tensile strength, ductility and yield strength values, corresponding to the increased mechanical properties of the silica particle-reinforced composite material. A method of preparing a polypropylene/acrylonitrile–butadiene–styrene/C18-functionalized silica composite that can be processed using FDM 3D printing was also described [21]. The result was an activated, 3D-printed object with a porous structure that allows access to the silica particles while maintaining their macroscopic size and shape. The 3D-printed device is dedicated to use in the solid phase microextraction (SPME) procedure.

Minerals are equally widely used as an additive to plastics, mainly due to their low cost and simple processing. An example of such a mineral is bentonite—a naturally occurring clay mineral with very small particle sizes and a high degree of plasticity [22]. Studies showed that the introduction of an additive to the polymer matrix has a positive effect on the thermal and mechanical properties of the composite, such as hardness, tensile strength and elongation [23,24,25,26].

Currently, additive manufacturing techniques are an increasingly common method of producing three-dimensional elements based on a computer-generated model. These techniques based on melt extrusion are very interesting, and the range of available polymer raw materials is impressive. However, the polymer materials described so far used in rapid prototyping technologies using molten polymer extrusion methods are based only on the basic, standard, most available plastics.

Therefore, as part of the work, polymer composites dedicated to additive manufacturing applications were developed and produced. Fillers were selected and introduced into the matrix of standard plastic. A detailed characterization of the impact of selected fillers on the functional properties of the polymer matrix-polycarbonate (PC), including rheological, mechanical, structural and physicochemical properties, allowed for an increase in the range of polymer composites and their use in rapid prototyping technologies, as well as supplementing the literature on databases regarding the characteristics of the obtained materials.

## 2. Materials and Methods

### 2.1. Materials

Commercially available polycarbonate (Transparent, Polylite™, Shanghai, China) (marked as PCF) was used as the polymer matrix. The PCF filling consisted of the following: silica modified with aluminum oxide, marked with the symbol S (Aerosil MOX 170, Evonic Industries, Hanau, Germany), and bentonite modified with a quaternary ammonium salt (BAR-QUAT^®^ DM80, Lonza, Switzerland) (product technical “Specjal”, Zębiec SA Zakłady Górniczo-Metalowe, Zębiec, Poland), which was marked with the symbol B in the work. Data regarding the method of obtaining B were described and patented earlier [5,6].

Fillers were introduced into PCF to investigate the impact of their presence and quantity on changes in the functional properties of the material, in particular fluidity/viscosity, mechanical properties and thermal stability of the material. The same amount of additives was used as in our previous works [27,28,29,30].

Each composition included a compatibilizer (C) to better mix the ingredients; chemically modified polyethylene grafted with maleic anhydride with the trade name Fusabond E926 (DuPont, Wilmington, DE, USA) was used.

The compositions of the developed compositions are summarized in Table 1.

### 2.2. Sample Preparation

Appropriately prepared (dried in a vacuum dryer (SPT-200, Colect, Kraków, Poland); PCF (100 °C; 4 h); S and B (100 °C, 24 h)) components of the composition were homogenized using a twin-screw extruder (Coperion, ZSK 18ML, Stuttgart, Germany) equipped with a granulation line. The assumed process parameters were as follows: extrusion speed 400 rpm, extrusion efficiency 4 kg/h and temperatures of individual zones in the range of 220–250 °C.

From the composites produced in this way, filaments with a diameter of 1.75 ± 0.05 cm were produced using a designed line for obtaining filaments made according to the design by Gamart S.A. (Łańcut, Poland). The view of the mentioned line was included in previous works [27,28,29,30]. The parameters used were as follows: engine speed 180 rpm, extraction speed 193 mm/s, filament placement speed on the spool 154 mm/s, temperature of individual zones in the range of 215–240 °C.

The samples were obtained on a TierTime Up Box+ printer (Beijing, China) using MEM technology. Optimal printing parameters were selected in accordance with the literature reports.

**Figure 1:**
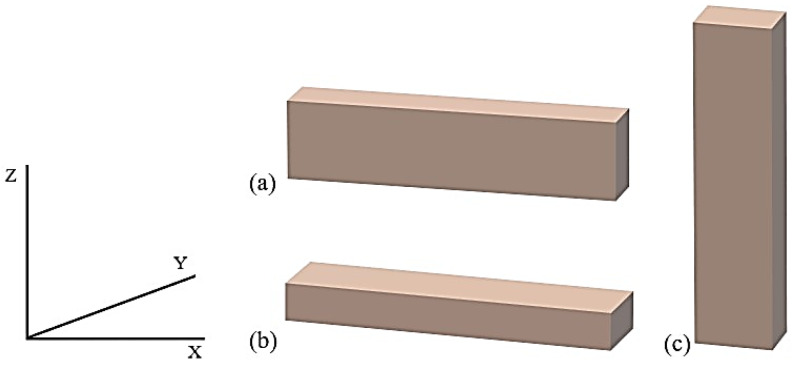
Print orientation view, in order: (**a**) edge (Y), (**b**) horizontal (X), (**c**) vertical (Z).

The orientation of the structure of the element manufactured in 3D has a significant impact on the mechanical and functional properties [31,32,33,34,35]. The detail should be placed in such a way as to ensure good stability and proper adhesion to the work platform during the process (Figure 1).

The most frequently used arrangement is edge and flat orientation because they allow for obtaining very good mechanical properties [32,33,34], often twice as high as in the vertical orientation [35]. Elements obtained in the edge orientation show the highest tensile strength and elongation at break, and Young’s modulus is similar to that obtained in the horizontal configuration. The *X*-axis arrangement ensures the highest bending strength and the highest impact strength [32,34].

**Figure 2:**
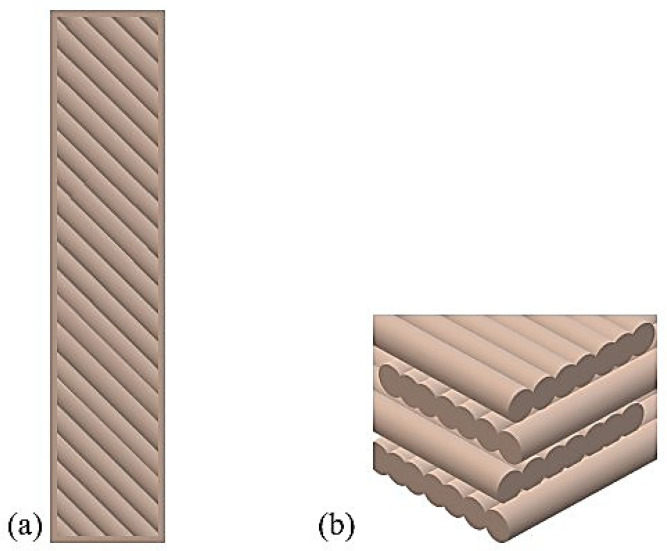
Raster orientation: (**a**) 45°, (**b**) −45°/+45°.

Raster orientation (Figure 2) in FFF/MEM/MEP technologies is an important parameter because it affects the strength, dimensional accuracy and quality of the surface finish of the details.

Due to good mechanical properties, especially very good tensile strength, raster angles of 0° and 45° are often used. The 0° fiber orientation angle allows for the highest strength (fiber orientation in the direction of the applied forces), up to 25% higher compared to the 90° orientation [32,33,34,35,36]. Moreover, the configurations also ensure very good fatigue resistance of the manufactured details; the best results are achieved with a 45° raster orientation [37,38]. Additionally, the 45° fiber arrangement ensures very good impact resistance, which is why it is a regularly used variant [37,38]. In order to obtain good mechanical properties, cross-printing orientations of 0°/90° and −45°/+45° are most often used [39,40].

The most important parameter influencing the mechanical properties is the filling density of the details [41,42,43]. The load-bearing capacity of the part increases with the increase in the amount of material inside the element; therefore, the best results of mechanical and functional properties are obtained by using 100% filling [44,45]. The part manufactured in this way guarantees a compact, solid structure, but this is also directly related to the longer process duration. Sometimes, scientists use lower infill density and different infill patterns to shorten printing time or save material [46,47].

The layer height is another 3D printing parameter that can be appropriately selected depending on the expected process results. A smaller layer height will result in an increase in their number and, thus, an increase in the process duration, which will significantly affect the properties of the manufactured details [48]. A given larger number of layers will cause a large temperature gradient towards the first one, which is directly related to the distortions occurring between the layers and within them. For printed details, an increase in layer height causes a decrease in tensile strength (increases stiffness) and an increase in bending strength [36,49]. The specified lower layer thickness causes the extruded molten filament to be compressed between the existing layer and the nozzle or printer build plate. In this way, the cylindrical fiber deforms plastically, and its cross-section changes from round to more oval, increasing the contact and wetting surface, which results in a more favorable bonding of adjacent fibers [50]. The optimal and most frequently used layer height is 0.2 mm [39,51,52,53].

The mechanical properties of elements obtained using FFF/MEM/MEP technologies are also influenced by the diameter of the nozzle used. A larger nozzle diameter ensures easier distribution of the molten polymer material. By increasing the ratio of the nozzle diameter to the layer thickness, it is possible to improve the mechanical strength of the part, as it increases the contact area between the layers [53,54]. By simultaneously controlling the nozzle size and layer thickness, the air gap between adjacent plastic fiber strands can be controlled.

The process temperature is a very important factor influencing the mechanical and functional properties of the parts but also allowing the optimization of the surface finish of the elements [55]. A change in temperature has a direct impact on the fluidity of the material, which results in a change in surface roughness. The use of higher temperatures (but still within the plastic processing temperature) may result in better surface quality [56]. At higher temperatures, the material solidifies slower, so it is possible to obtain a smooth surface. Moreover, an increase in temperature causes a decrease in the viscosity of the material, which is why it is possible to smooth the surfaces of the ends of individual layers (steps) but also to increase the bonding strength, which results in obtaining details with improved mechanical properties [56,57]. The lower temperature negatively affects the obtained surface quality but ensures better dimensional accuracy and allows for easy removal and detachment of the element from the work table after the process [56,57].

In accordance with the above guidelines, the optimal parameters of the 3D printing process were selected, which are summarized in Table 2.

For comparative tests, shapes with the same dimensions were obtained using injection molding on an injection molding machine (Haake MiniJet II, ThermoScientific, Waltham, MA, USA).

Selected injection molding parameters are presented in Table 3.

**Figure 3:**
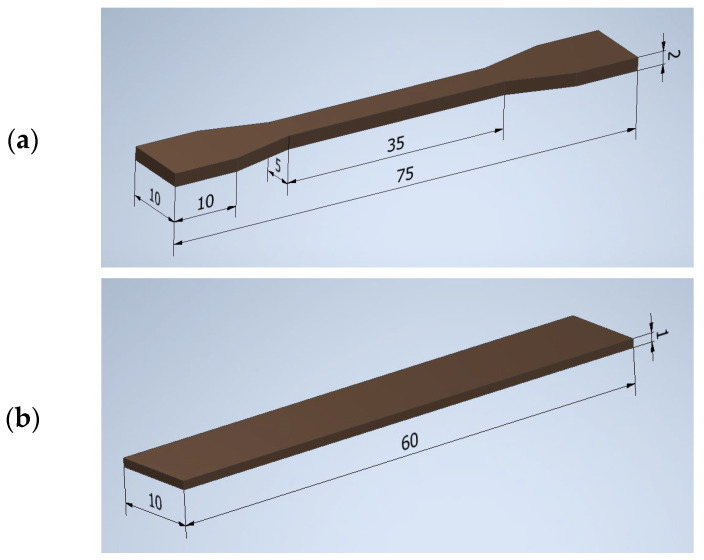
Obtained samples: (**a**) paddle, (**b**) bar.

The dimensions and shape of the obtained samples are shown in Figure 3.

### 2.3. Methods

Mass melt flow rate, MFR, was determined using a plastometer (DYNISCO 4781, Kayeness Inc., Honey Brook, PA, USA). The plastometer was heated to the temperature appropriate for the tested materials (250 °C), and then samples weighing approximately 4 g were introduced. The measurement was carried out using a load of 2.16 kg; the sample removal time was 15 s. The test was made in accordance with [58].

The rheological properties were varied by determining the viscosity of the tested materials. The determination was performed using a capillary rheometer (Smart RHEO, Instron Ceast, MA, USA). The rheometer was equipped with a capillary 40 mm long and 1.16 mm wide, heated to a temperature appropriate for the tested materials (250 °C), then samples weighing approximately 10 g were introduced, which were thermostated for 300 s under the initial piston load. The appropriate shear rate range was 100 1/s–3000 1/s. The test was made in accordance with [59].

Rockwell hardness was measured using a hardness tester (Zwick/Roell, Zwick GmbH & Co., Ulm, Germany). Ten determinations were made for each series of materials in accordance with [60].

Strength properties were determined during a static tensile test using a testing machine (INSTRON 5967, Grove City, PA, USA). Young’s modulus was measured at a tensile rate of 5 mm/min (up to 1% tensile strain), after which the tensile rate was increased to 50 mm/min. The test with an extensometer was carried out in the range of elastic strains (to obtain 1% tensile strain). The test was conducted at ambient temperature. The test was made in accordance with [61].

SEM, a scanning electron microscope (Hitachi TM3000, Red Star Vietnam Co., Hanoi, Vietnam) equipped with a microanalysis device with energy dispersive spectroscopy (EDS), was used to observe the structure of the obtained polymer composites. The sample was properly prepared by placing it in liquid nitrogen and subsequently obtaining a brittle fracture by impact fracture. Before observations were made, the samples were sputtered with a layer of gold and palladium.

The next analysis performed was thermogravimetric analysis, TGA, which was performed using a TGA/DSC 1 apparatus (Mettler Toledo DSC 1 Star^®^ System, METTLER Toledo, Schwerzenbach, Switzerland). Samples weighing approximately 5 mg were placed on platinum plates and heated in the temperature range from 25 °C to 600 °C at a rate of 10 °C/min. The test was conducted in a nitrogen atmosphere.

Differential scanning calorimetry, DSC, was also performed using a TGA/DSC 1 device (Mettler Toledo DSC 1 Star^®^ System, Schwerzenbach, Switzerland). Samples (each weighing approx. 6 mg) were placed in sealed aluminum crucibles, then heated in the temperature range from −90 °C to 300 °C at a rate of 10 °C/min, cooled to −90 °C at a rate of 10 °C/min and then heated again to 300 °C at a rate of 10 °C/min. Measurements were performed in a helium atmosphere.

The last measurement taken was WAXS, wide-angle X-ray diffraction. The determination was performed using a diffractometer (NanoStar-U, Bruker Inc., Billerica, MA, USA), which was equipped with a two-dimensional detector in transmission geometry. The possible range of the scattering angle was from 0° to 28°.

## 3. Results and Discussion

The rheological data of materials are important from the point of view of proper design and the proper conduct of processing processes. The fluidity of materials is very important in terms of processing because materials with higher fluidity will more easily fill the entire volume of the injection mold and will also allow for the denser printing of elements made using 3D printing technology.

In order to investigate the influence of the fillers used on the fluidity of the PCF-based composites, the mass flow rate was determined, and the results obtained are summarized in Table 4.

Analyzing the results obtained for PCF-based composites, it was observed that the introduction of filler B significantly improved the fluidity of the unmodified polymer. The introduction of modified bentonite in an amount of 3% allowed for obtaining a result that was 13.21% higher compared to the result obtained when using 1.5%B. A similar relationship was obtained after introducing 1%S into the polymer matrix (the result was 0.65% higher than that obtained for PCF). In this case, the result of the mass flow rate index was 3.30% higher compared to the composite containing 0.5% of the additive.

The literature data indicate that composites with the addition of mineral fillers show increased mass flow rates when the filler is introduced to an amount of 3% by weight. It was reported that the introduction of larger amounts of these fillers may adversely affect MFR results [39,62,63].

In the literature, rheological properties are most often presented by describing only the MFR value and the mass flow rate index, and less often, they are supplemented by presenting the viscosity value.

**Figure 4:**
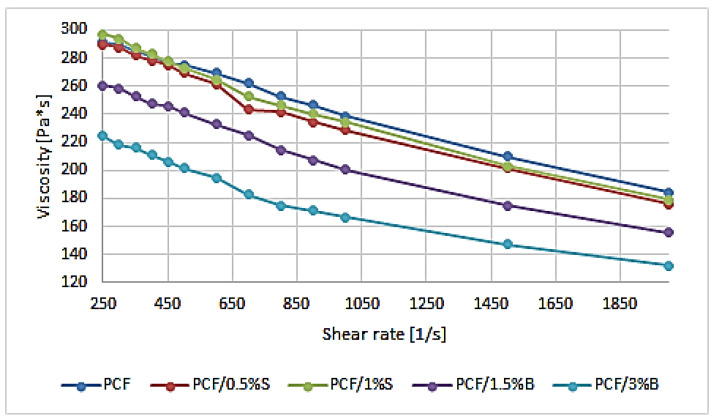
Viscosity curves of PCF polymer and composites based on PCF.

Based on the obtained test results, it was observed that the viscosity of individual composites decreases as the shear rate increases (Figure 4). The introduction of modified fillers into the polymer matrix resulted in a significant change in the viscosity of the obtained composites, especially after the introduction of filler B. PCF, PCF/0.5%S and PCF/1%S obtained similar flow curves at low shear rate values, obtaining a difference in the results at e.g., 800 s^−1^ 238.33 Pa·s, 228.58 Pa·s and 234.40 Pa·s, respectively. The lowest viscosity was obtained for the PCF/3%B composite and then PCF/1.5%B, which, at a speed of 800 s^−1^, achieved viscosity lower by 26.69% and 10.09% compared to the polymer. The phenomenon can be explained by the lamellar structure of bentonite, which allowed for obtaining higher values of the mass flow rate index, which directly affects the presented viscosity results [64]. At higher shear rates, the differences between the polymer and composites filled with modified silica or modified bentonite remain unchanged on average.

Most often, 3D printing technology is compared to injection molding. The main difference is the mechanical strength of prints. It is approximately 30% of the mechanical strength of a detail made of the same material produced by injection. The literature data confirmed the obtained results [20,65,66] and directly linked the phenomenon to the structure of the samples made; samples obtained in the injection process are more homogeneous because the additive manufacturing process creates spaces between the material threads [67]. For this reason, results obtained using different techniques are most often presented separately. It should also be noted that no post-processing-surface smoothing was applied to the 3D-printed samples. The existing surface roughness had no impact on the obtained results of performance tests, and the surface quality was not the subject of this study.

**Figure 5:**
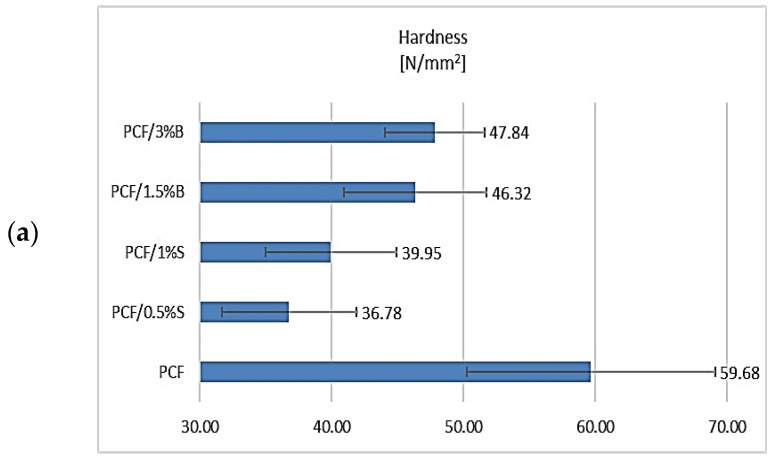

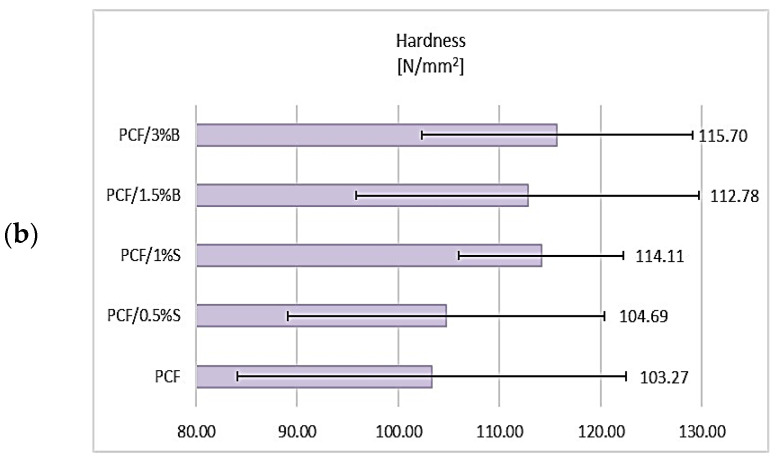
Hardness test results: (**a**) samples obtained by 3D printing, (**b**) samples obtained by injection molding.

In the next stage of work, PCF and composites based on it were tested for hardness, and the results are presented in Figure 5. The obtained results of the hardness of shapes made using the additive manufacturing technique (Figure 5a) allowed for concluding that the introduced fillers had a negative impact on the hardness of the obtained composites. The lowest result was obtained for the PCF/0.5%S composite, exactly 22.90 units lower compared to the polymer. Better results were obtained when using higher concentrations of 1%S and 3%B fillers. Comparing the results obtained with those presented in Figure 5b, it was noticed that the introduced fillers increased the hardness of the shapes obtained by injection molding, regardless of the additive concentration. Higher hardness was obtained for the composites compared to the unfilled polymer. The highest result was obtained for the composite containing 3%B (115.70 N/mm^2^).

The static tensile test is the basic method for testing the strength properties of polymers and composite materials.

When assessing the results listed in Table 5 for shapes produced using rapid prototyping technology for PCF and polycarbonate-based polymer composites, it was noticed that the introduced modified fillers increased the elasticity of the material. The observations confirm the previously presented and discussed results because the composites obtained lower hardness compared to the polymer. The introduction of modified silica caused a decrease in Young’s modulus by 10.02% at the content of 0.5% S and at 1% S by 8.64% compared to the polymer. The lowest modulus value was obtained for the PCF/0.5%S material, which is 1661.02 MPa. The addition of modified bentonite (B) did not significantly affect the obtained determination results.

However, the analysis of the results obtained for shapes obtained by injection molding showed a positive effect of the addition of modified fillers on Young’s modulus. An increase in the stiffness of the composites compared to polycarbonate was observed, up to 7.41% for PCF/3% B. Similar conclusions were described in the analysis of hardness results (Figure 5). For shapes obtained by 3D printing, a decrease in tensile stress results was obtained ranging from 5.43% to 11.21%, and the strain at break was also reduced by 8.06% to 19.85% compared to the polymer matrix. The lowest tensile stress at break was observed for PCF/3% B, while the lowest strain at break was observed for PCF/1.5% B. Analyzing the results of mechanical tests for shapes obtained by injection molding, it was found that the presence of modified fillers in the polymer matrix resulted in a reduction in tensile stress. However, the observed slight decrease in stress does not exceed 4.22%. However, the decrease in strain at break for all tested composites ranges from 22.62% to 58.09%, where the lowest value of strain at break (13.75%) was obtained for PCF/1% S.

**Figure 6:**
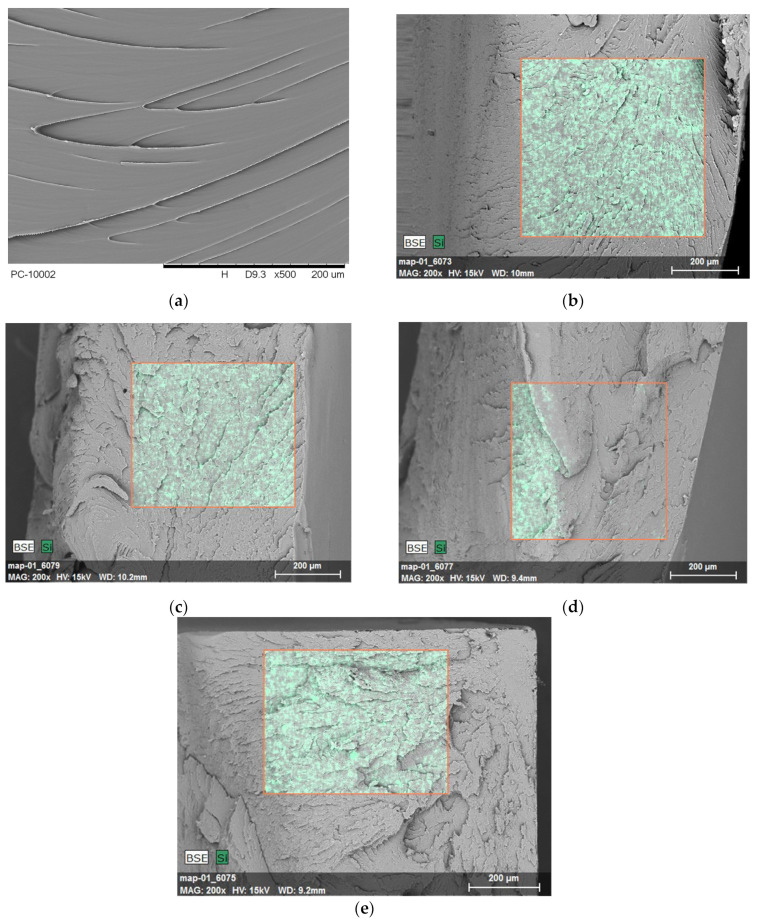
SEM/EDS imaging results of PC filament polymer and PCF-based composites: (**a**) PCF, (**b**) PCF/0.5% S, (**c**) PCF/1% S, (**d**) PCF/1.5% B, (**e**) PCF/3% B. The orange outline marks the area subjected to EDS analysis, which was performed to observe the degree of filler dispersion and the distribution of the silicon element.

SEM, a scanning electron microscope, with EDS, a device for microanalysis of chemical composition, was used to analyze the brittle fracture morphology of the obtained polymer composites. The sample fractures were obtained after immersing them in liquid nitrogen and subsequent impact shattering. The results were collected and presented in Figure 6.

Based on SEM micrographs of brittle fractures of the tested PCF-based composites, it was observed that the brittle fracture of PCF presents a smooth surface (Figure 6a). The introduction of fillers S and B resulted in the formation of more torn plates; it is difficult to distinguish the phases present (polymer/filler); therefore, the EDS system was used (Figure 6b–e), which allows determining the elemental composition of the composite surface layer and dispersing the filler. The element silicon was selected for SEM/EDS analysis because all selected fillers contain it in their chemical structure. The selected research area is marked in red. The analysis of the examined area showed adequate dispersion of the introduced modified fillers, and moreover, no clusters or agglomerates of additives were observed. Similar regularities can be seen in the results of B. Pilica et al. [68], which indicates that the presence of a small amount of about 1% by weight of silica in the polymer is characterized by good and uniform dispersion. However, the confirmation of appropriate dispersion for composites filled with bentonite in an amount of up to 3% by weight was found elsewhere [69]. The observations confirm well-selected conditions for the homogenization of the ingredients.

**Figure 7:**
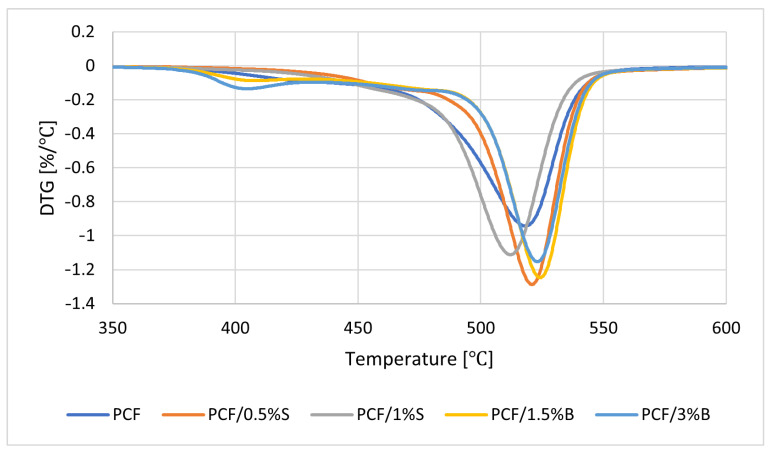
Results of DTG analysis (mass change derivative curve) of PCF polymer and composites based on PCF.

The obtained results of TGA, thermal stability, PCF and PCF-based composites tests are summarized in Figure 7 and Table 6. Several parameters were determined, including the temperature of 5% mass loss (T_5%_), the beginning of the degradation process as well as the maximum temperature of the degradation stages (T_max_).

The unmodified polymer is characterized by high thermal stability, as PCF decomposition begins at 449–450 °C (Figure 7 and Table 6). The introduced silica additive increased the thermal stability of the material by 23 °C for PCF/0.5% S and 14 °C for PCF/1% S, respectively (Table 6). Thus, a favorable interfacial interaction between the fillers used and the PCF polymer matrix was confirmed [70]. The reason may be the relatively large specific surface area of the silica filler. The introduction of silica, S, into the polymer matrix leads to the formation of an interlayer zone on the filler surface and, consequently, to the immobilization of polymer chains [71]. PCF, PCF/0.5% S and PCF/1% S are characterized by a one-stage thermal decomposition; however, the introduction of modified bentonite B into the polymer matrix resulted in a two-stage degradation of the materials (Figure 7). The presence of B in the polymer matrix decreased the thermal stability of polycarbonate (Table 6).

**Figure 8:**
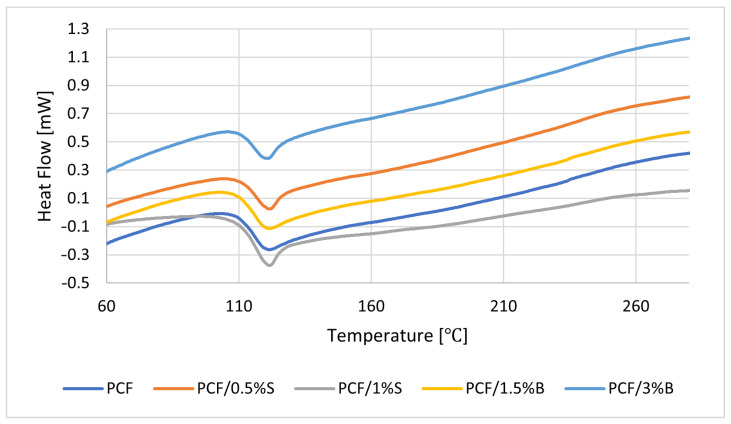
Results of differential scanning calorimetry (DSC) analysis of PCF-based composites.

In the next stage of work, a DSC analysis of polycarbonate and its composites was carried out (Figure 8). An inflection appears in the thermograms at approximately 125–130 °C, characteristic of the glass transition temperature of the PCF phase. The literature data confirm the obtained result [70,72]. To sum up, no influence of the type, modification of fillers and their dispersion in the polymer matrix on the change in glass transition temperature was observed.

The morphology and molecular orientation of the composites and fillers were characterized using WAXS analysis.

**Figure 9:**
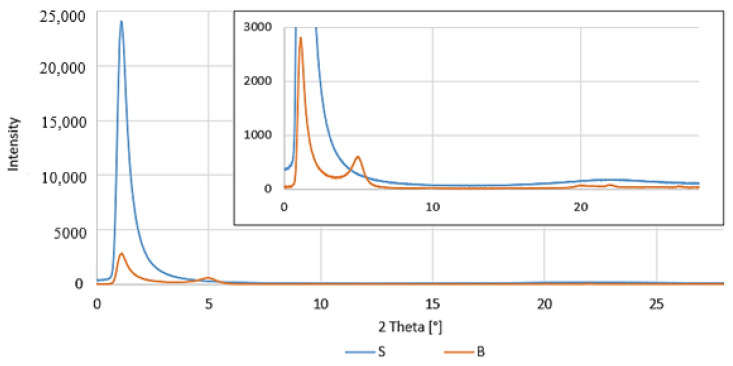
WAXS patterns: fillers.

**Figure 10:**
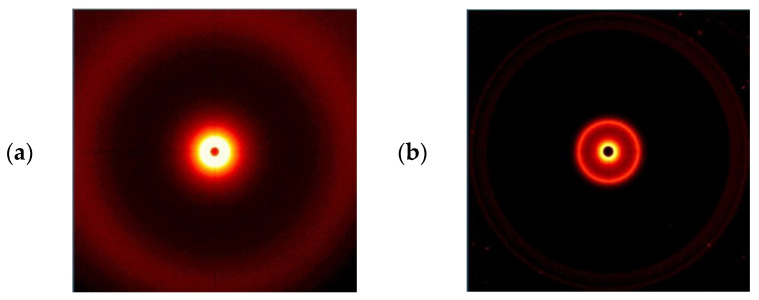
Results of 2D WAXS imaging of the used fillers: (**a**) S, (**b**) B.

Graphs of the radiation intensity as a function of the scattering angle of the fillers are shown in Figure 9, and wide-angle 2D X-ray diffractograms are shown in Figure 10.

The possible range of the scattering angle of the tested materials was from 0° to 28°; therefore, only peaks were observed in the curves for one filler-modified bentonite, B (Figure 9). Under these conditions, it was not possible to observe modified silica, S, although its presence in this range was noticed in 2D-WAXS images (Figure 10a), but the obtained intensity was too low to register the scattering angle.

The distance between successive planes of the filler (d_hkl_) was calculated from the Bragg formula:

(1) $$d_{\mathrm{hkl}}=\frac{n\lambda }{2sin\theta },$$

where n is the degree of diffraction (*n* = 1, 2…), λ is the wavelength of radiation used and 2θ is the angle at which the diffractive peak occurs, as read from the WAXS graph.

The particle size in the Scherrer formula was also determined:

(2) $$D_{\mathrm{hkl}}\;=\frac{K\lambda }{bcos\theta },$$

where D_hkl_ is the reflex width dependent on the size of crystallites, K is Scherrer’s permanent, K = 1, λ is the wavelength of radiation used and b is the half-width of the diffraction peak for the plane (_hkl_).

Analyzing the obtained WAXS results measured for modified fillers (Figure 9), one peak is visible at 4.98°, which, according to the literature, can be attributed to diffraction reflection from bentonite (001) sheets [73]. The distances between subsequent packages of filling plates, d_khl_ (for B, this is 18.20 Å), and the size of their particles, D_khl_ (for B, this is 110.8 Å), were calculated.

**Figure 11:**
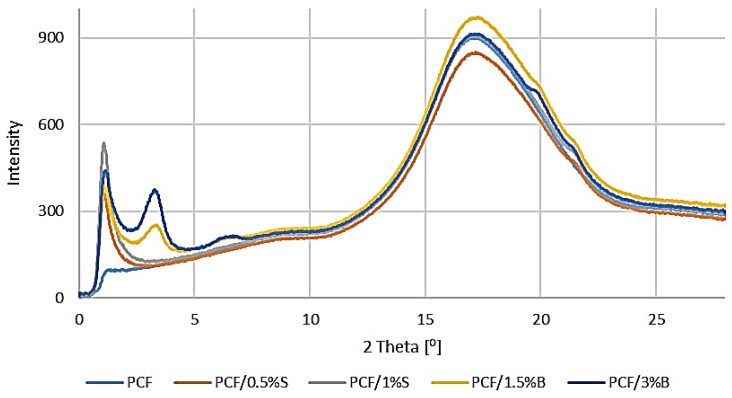
WAXS patterns: PCF and composites with the addition of modified S and B fillers.

Analyzing the obtained test results, it was found that PCF-based composites are characterized by the occurrence of one intense peak at 2θ of approximately 17°, which can be assigned to the PC band (Figure 11). The introduced modified silica was well dispersed in the polymer matrix. However, composites containing modified bentonite are characterized by the occurrence of two additional peaks at the 2θ value of approximately 3.18° and 6.29°. In order to determine the filler dispersion, the distances between successive packets of B plates and their particle sizes were calculated. For B, d_khl_ is 28.49 Å, while D_khl_ is 70.78 Å. Comparing the obtained d_khl_ and D_khl_ results with those calculated for the filler, an increase in the distance between the bentonite plate packets by 10.29 Å and a decrease in the particle size by 40.02 Å was observed. It can, therefore, be concluded that in PCF/1.5% B and PCF/3% B, the bentonite layers were pulled apart but not fully dispersed.

## 4. Conclusions

The influence of the content and amount of fillers, such as silica modified with aluminum oxide and bentonite modified with a quaternary ammonium salt, on the functional properties of a commercially available filament made of a traditional plastic such as polycarbonate was determined. As part of the work carried out, modern polymer materials were obtained dedicated to 3D printing applications, especially in FDM/FFF/MEM technologies. Selected functional properties of the new composites were examined, including their fluidity; viscosity; mechanical properties, such as hardness and parameters determined during the static tensile test, and thermal stability, and their structure was determined using a scanning electron microscope and wide-angle X-ray diffraction. It was found that the addition of B significantly increased the fluidity of the polymer; the introduction of a filler in the amount of 1.5% allowed us to obtain a result that was 6% higher compared to PCF, while the amount of 3% was 20% higher. However, the presence of filler S in the polymer matrix did not affect the obtained results of the mass melt flow rate. The obtained MFR results were confirmed by determining the viscosity of the produced polymer composites. PCF/1.5% B and PCF/3% B are characterized by the lowest viscosity among the tested materials. Satisfactory results of mechanical properties were obtained; among others, it was found that the introduced modified fillers increased the elasticity of the material. The introduction of modified silica caused a decrease in Young’s modulus by 10.02% at the content of 0.5% S and at 1% S by 8.64% compared to the polymer. The lowest modulus value was obtained for the PCF/0.5% S material, which is 1661.02 MPa. Moreover, the introduced modified S filler significantly increased the thermostability of polycarbonate by 23 °C for PCF/0.5% S and 14 °C for PCF/1% S, respectively. The SEM and WAXS results confirmed the appropriate dispersion of fillers in the polymer matrix, which proves well-selected process conditions for the homogenization of components and subsequent sample production. The work proves that the use of polymer composites can unlock the efficiency of additive manufacturing and may affect basic problems resulting from the characteristics of the process, such as printability (fluidity and viscosity) of the material. The databases do not contain full characteristics of the functional properties of materials dedicated to 3D printing, and if they exist, they concern unmodified, standard polymer materials. That is why it is so important to constantly search for new material systems and precisely characterize their features.

## Figures and Tables

**Table 1:** Compositional data of the composites.

Composition	PCF Content (wt.%)	S Content (wt.%)	B Content (wt.%)	C Content (wt.%)
PCF	100.0	-	-	-
PCF/0.5% S	98.5	0.5	-	1.0
PCF/1% S	98.0	1.0	-	1.0
PCF/1.5% B	97.5	-	1.5	1.0
PCF/3% B	96.0	-	3.0	1.0

**Table 2:** Three-dimensional printing parameters.

Parameter	Nozzle Diameter [mm]	Layer Height [mm]	Filling [%]	Fill Pattern	Extrusion Temperature [°C]	Worktable Temperature [°C]	Print Speed [mm/s]
Value	0.4	0.2	100	45°/+45°	250	90	70

**Table 3:** Injection molding process parameters.

Parameter	Injection Temperature [°C]	Mold Temperature [°C]	Injection Time [s]	Plasticizing Time [s]	Injection Pressure [bar]	Post-Injection Pressure [bar]
Value	250	70	5	120	paddles	
					750	700
					bars	
					850	800

**Table 4:** Compositional data of the composites.

Composite	PCF	PCF/0.5%S	PCF/1%S	PCF/1.5%B	PCF/3%B
MFR	16.8 ± 0.17	16.37 ± 0.23	16.91 ± 0.02	17.87 ± 0.49	20.23 ± 0.06

± standard deviation.

**Table 5:** Summary of the results of the mechanical properties of PCF material and the obtained composites based on it.

Composition	Young’s Modulus [MPa]	Stress at Break [MPa]	Strain at Break [%]	Young’s Modulus [MPa]	Stress at Break [MPa]	Strain at Break [%]
	3D Printing			Injection		
PCF	1846.00 ± 29.54	67.27 ± 5.03	6.70 ± 0.16	1978.04 ± 87.41	56.09 ± 0.29	32.81 ± 20.22
PCF/0.5%S	1661.02 ± 37.25	60.21 ± 0.63	5.69 ± 0.62	1988.33 ± 8.16	54.63 ± 2.31	17.42 ± 3.53
PCF/1%S	1686.59 ± 56.41	60.93 ± 1.53	6.16 ± 0.48	2044.90 ± 6.03	56.58 ± 1.86	13.75 ± 6.56
PCF/1.5%B	1799.36 ± 1.93	63.62 ± 1.13	5.37 ± 0.14	2046.66 ± 18.27	55.83 ± 0.99	21.78 ± 9.83
PCF/3%B	1747.75 ± 21.53	59.73 ± 0.16	5.82 ± 0.36	2124.70 ± 33.76	53.72 ± 2.87	16.51 ± 10.92

± standard deviation.

**Table 6:** The results of research on the properties of thermostability of composites.

Composites	T_2%_ [°C]	T_5%_ [°C]	T_1_ [°C]	ΔV_1_ [%/°C]	T_2_ [°C]	ΔV_2_ [%/°C]	R_600_ [%]
PCF	418.07	449.41	524.77	0.94	-	-	27.04
PCF/0.5%S	446.07	472.64	525.99	1.29	-	-	29.29
PCF/1%S	437.83	464.10	518.47	1.11	-	-	27.61
PCF/1.5%B	409.74	445.70	406.04	0.09	529.85	1.25	27.15
PCF/3%B	400.95	424.67	404.65	0.13	528.82	1.15	26.81

## Data Availability

Data are contained within the article.
